# Artificial Intelligence in Non-Surgical Cosmetic Procedures: A Multi-Stakeholder Revolution

**DOI:** 10.1007/s00266-026-05689-3

**Published:** 2026-03-19

**Authors:** Jiancheng Li, Youyou Li, Jianquan Yan, Lijun Yan, Qin Li

**Affiliations:** 1 Guangdong University of Technology, Guangzhou, China; 2 Communication University of China, Beijing, China; 3 Chinese Association of Plastics and Aesthetics, Beijing, China; 4 AIST Medical Aesthetic Group, 17/F, Block B, Chenghua Development Building, No.98, Shuangcheng 2nd Road, Chenghua District, Chengdu, Sichuan China

**Keywords:** Artificial intelligence, Machine learning, Cosmetic, Personalized medicine, Non-surgical procedures

## Abstract

Non-surgical cosmetic procedures have experienced significant growth, driven by advancements in technology and changing consumer demographics. The global non-surgical aesthetic market is projected to reach $90.2 billion by 2030, with Asia, particularly China, being a major contributor. Artificial Intelligence (AI) is revolutionizing these procedures by enhancing patient engagement, clinical decision-making, and operational efficiency. AI applications span from personalized aesthetic assessments and treatment planning to real-time monitoring and post-procedural care. This review synthesizes recent advancements in AI integration across the non-surgical cosmetic workflow, addressing challenges such as data privacy, algorithmic bias, and the need for comprehensive physician training. By adopting a multi-stakeholder perspective, the review highlights AI’s potential to democratize access to personalized, safe aesthetic care while emphasizing the importance of ethical considerations and regulatory frameworks.

*Level of Evidence IV* This journal requires that authors assign a level of evidence to each article. For a full description of these Evidence-Based Medicine ratings, please refer to the Table of Contents or the online Instructions to Authors www.springer.com/00266.

## Introduction

In contemporary society, cosmetic products have become an indispensable aspect of human well-being and quality of life. It is evident that non-surgical cosmetic procedures are undergoing accelerated development in recent years. As demonstrated by the findings of research studies in this field, the global non-surgical aesthetic market was valued at $53.2 billion in 2023. Projections indicate that this figure will increase to $90.2 billion by 2030, at a compound annual growth rate (CAGR) of 7.2% [1]. The Asian market accounts for 42 percent of the global non-surgical aesthetic market, with China being the largest single market in Asia. The growth rate of China’s light medical aesthetics market was 18.6% from 2020 to 2025, which is significantly higher than the global average [2].

This cosmetic non-surgical field has exhibited a tendency toward parallel development of technological diversification and market scale. The treatment is represented by injectable fillers (e.g., hyaluronic acid, collagen), neuromodulators (e.g., botulinum toxin), photoelectric treatments (e.g., lasers and radiofrequency), and minimally invasive skin management (e.g., chemical skin resurfacing and mesotherapy), with characteristics of low trauma, fast recovery, and reversible effects. And substantial structural shifts have been observed within the consumer demographic. Young women aged 18–35 constitute over 70% of the total, while the proportion of male beauty seekers has increased from 12% in 2019 to 23% in 2023. The prevailing demands encompass facial rejuvenation, contouring, and skin texture enhancement [3].

However, challenges persist in meeting the diverse needs of stakeholders, including patients seeking personalized outcomes, clinicians requiring precision-driven tools, and institutions aiming for operational efficiency et al. And cosmetic procedure needs to combine evidence-based scientific techniques and artistic understanding to help patients achieve desired aesthetic outcome [3]. As an artistic discipline, cosmetic procedures are inevitably subjective when measuring the success of aesthetic outcomes. Given these challenges, there is growing interest in the potential role of artificial intelligence (AI) in cosmetic.

The integration of artificial intelligence (AI) into non-surgical cosmetic procedures is revolutionizing workflows across pre-, intra-, and post-procedural stages. By leveraging advancements in computer vision, large language models (LLMs), and predictive modeling, AI addresses critical challenges in patient engagement, clinical decision-making, and operational efficiency, while aligning with the evolving demands of patients, clinicians, institutions, and regulatory bodies [4–6]. Despite this accelerating diversification, current evidence remains fragmented: most studies are small, heterogeneous, and technically oriented, with limited external validation and uncertain generalizability across skin types, ethnic groups, or clinical settings. On the other hand, the evolving demands of patients, clinicians, institutions, and regulatory bodies are sometimes not deeply understood by AI technicians, which leads to the gap between development and real-world practice.

The present review responds to this need by adopting a multi-stakeholder and function-oriented perspective on AI in non-surgical cosmetic procedures. Specifically, we pursue three objectives: (1) to map current AI applications across the non-surgical cosmetic workflow (pre-procedural assessment and counseling, intra-procedural guidance, and post-procedural monitoring); (2) to bridge the vision of cosmetic stakeholders and AI technicians, with a view to clarifying current cosmetic unmet needs and introducing how AI can possibly solve the issue; and (3) to identify the gap between technology development and real-world practice, and to determine the improvement factors of AI application. In consideration of the prevailing regional discord, we place a premium on the advancement and incorporation of AI at the local level, with a particular emphasis on aligning with regional demands, particularly in Asian populations where aesthetic preferences and anatomical features differ markedly from Western cohorts [7, 8].

## Methods

### Study Design and Reporting Framework

**Fig. 1:**
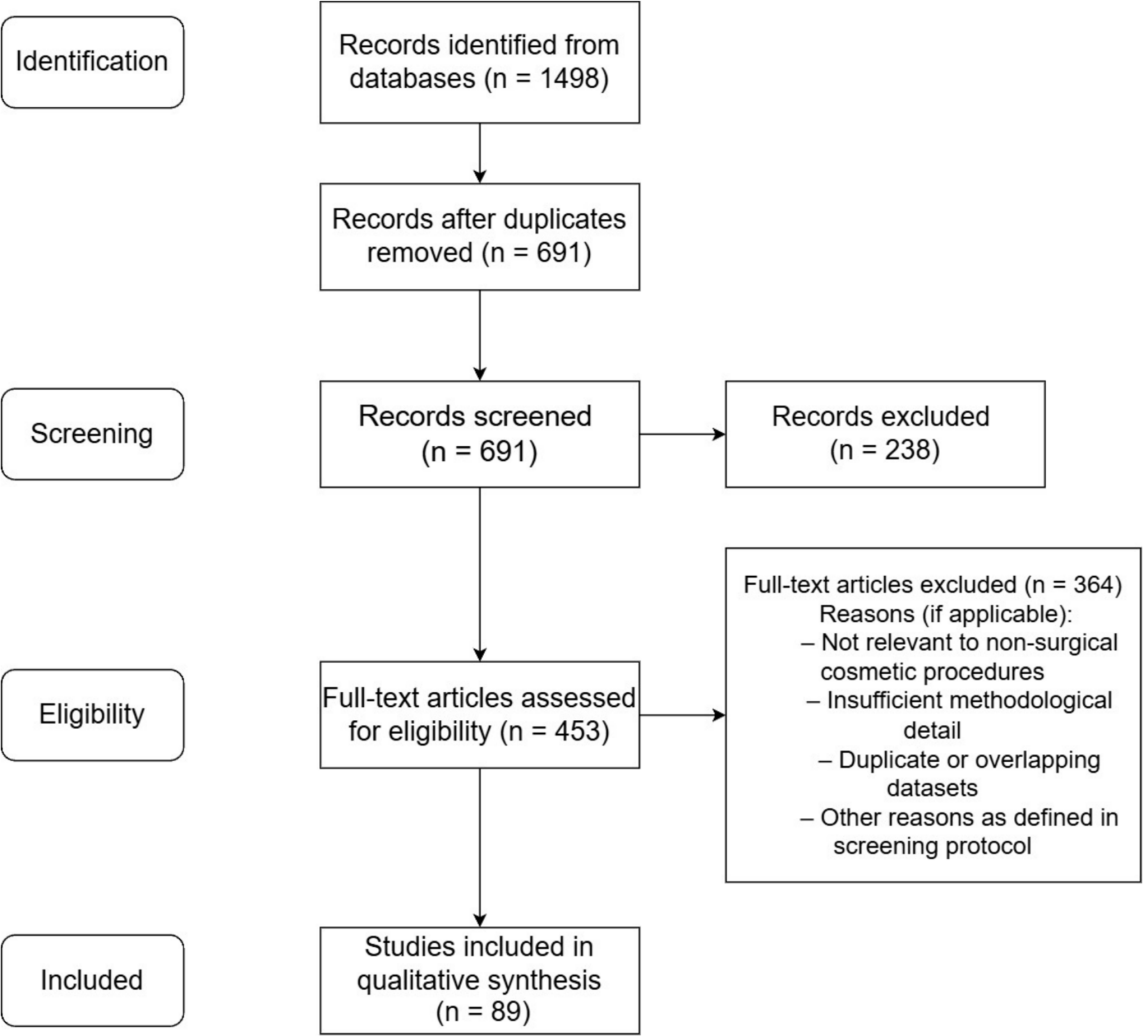
PRISMA flow diagram

We conducted a systematic review with narrative synthesis of AI applications in non-surgical cosmetic procedures. The review was designed and reported in accordance with the Preferred Reporting Items for Systematic Reviews and Meta-Analyses (PRISMA 2020) guidelines, insofar as these are applicable to predominantly qualitative and heterogeneous evidence. A PRISMA-style flow diagram (Fig. 1) summarizes the processes of record identification, screening, eligibility assessment, and inclusion.

Two reviewers (Reviewer A and Reviewer B) independently performed all stages of the review, including literature search, title and abstract screening, full-text review, and data extraction. Disagreements at any stage were first resolved through discussion; if consensus could not be reached, a third senior reviewer (Reviewer C) adjudicated. This process and the underlying decision rules were pre-specified before full-text screening commenced.

### Search Strategy

A comprehensive search of electronic databases was conducted to identify studies reporting the use or implications of AI in non-surgical cosmetic medicine. Four English-language databases (PubMed, Web of Science, Elsevier Scopus, and Google Scholar) and two Chinese-language databases (Wanfang Med Online and CNKI) were systematically searched.

We combined AI-related terms (for example, “artificial intelligence”, “AI”, “deep learning”, “machine learning”, “generative adversarial network*”, “neural network*”) with cosmetic-related terms (for example, “facial aging”, “facial rejuvenation”, “facial beauty”, “aesthetic*”, “cosmetic”, “skin aging”, “injectable*”, “botulinum toxin”, “filler*”, “laser”, “radiofrequency”). The search was limited to publications between January 2021 and December 2025 and to studies written in English or Chinese. Reference lists of included articles and related reviews were manually screened to identify additional relevant studies.

### Eligibility Criteria

We specified inclusion and exclusion criteria a priori.

Studies were eligible for inclusion if they met all of the following conditions. First, they reported on AI, machine learning, deep learning, computer vision, or related computational approaches applied either to non-surgical cosmetic procedures (for example, injectables, lasers, energy-based devices, skin management) or to facial and skin aesthetic assessment directly relevant to such procedures. Second, they involved human participants or human facial and skin data, such as clinical images or three-dimensional scans. Third, they provided sufficient methodological detail to understand the AI approach or its clinical integration, including information on data inputs, model type, and outcome measures. Fourth, they constituted original research articles, clinical studies, observational cohorts, algorithm development or validation studies, or systematic reviews and meta-analyses on these topics. Fifth, they were published in peer-reviewed journals or reputable conference proceedings. Finally, they were written in English or Chinese.

Studies were excluded if any of the following applied. First, they focused exclusively on surgical cosmetic procedures (for example, rhinoplasty and facelifts) without any non-surgical component or clearly transferable insights to non-surgical cosmetic medicine. Second, they reported only basic laboratory or animal experiments without direct human aesthetic outcomes. Third, they described purely technical AI developments (for example, generic facial recognition, surveillance, or identity verification systems) without explicit cosmetic or aesthetic relevance. Fourth, they comprised editorials, opinion pieces, letters, commentaries, or conference abstracts lacking substantive data or methods. Finally, they were duplicates, near-duplicates, or reports of the same dataset without novel analyses or outcomes.

### Study Selection and PRISMA Flow

The initial database search yielded 1,498 records. After removing duplicates, 691 unique records remained. Two reviewers independently screened titles and abstracts and excluded 482 records that were clearly irrelevant or did not satisfy the non-surgical aesthetic focus.

The remaining 209 full texts were assessed in detail for eligibility. Articles were excluded for the following main reasons: different primary focus, such as purely surgical applications (*n* = 202); insufficient methodological detail; non-peer-reviewed status; or overlap with other included studies (*n* = 73, including near-duplicate reports and redundant analyses).

Ultimately, 89 publications met all inclusion criteria and were included in the qualitative synthesis. Reasons for exclusion at each stage and the flow of records through the review process are presented in Fig. 1 (PRISMA diagram).

## Results

### Study Design Characteristics of Included Studies

Among the 89 included publications, study designs were heterogeneous. For descriptive purposes, we grouped them into five categories as defined in the Methods: algorithm development/technical validation, retrospective clinical studies, prospective observational or pilot studies, survey or questionnaire studies, and other designs (concept papers, expert opinion, and workflow reports). The majority of articles fell into the algorithm development/technical validation category, whereas only a minority provided prospective clinical data. The detailed distribution of study designs and corresponding references is summarized in Table 1.

**Table 1:** Distribution of study designs and corresponding references

Study design category	Number of studies, n	References
Algorithm development/Technical validation	37	[12, 14, 20, 24–26, 28–36, 38, 39, 41, 45, 48, 50, 56, 58, 63, 65, 69, 71, 72, 74, 75, 79, 81–83, 86, 104, 105]
Retrospective clinical study	5	[15, 27, 40, 64, 98]
Prospective observational/Pilot study	19	[16, 18, 19, 29, 37, 43, 47, 55, 56, 59, 62, 65, 66, 68, 78, 87, 89, 91, 103]
Survey/Questionnaire study	7	[13, 77, 84, 92–94, 108]
Other (review, concept, expert opinion, workflow etc.)	21	[11, 23, 42, 44, 46, 49, 51, 57, 60, 61, 73, 75, 76, 80, 85, 90, 97, 100–102, 106]

### Overview and Functional Taxonomy of AI Roles

**Fig. 2:**
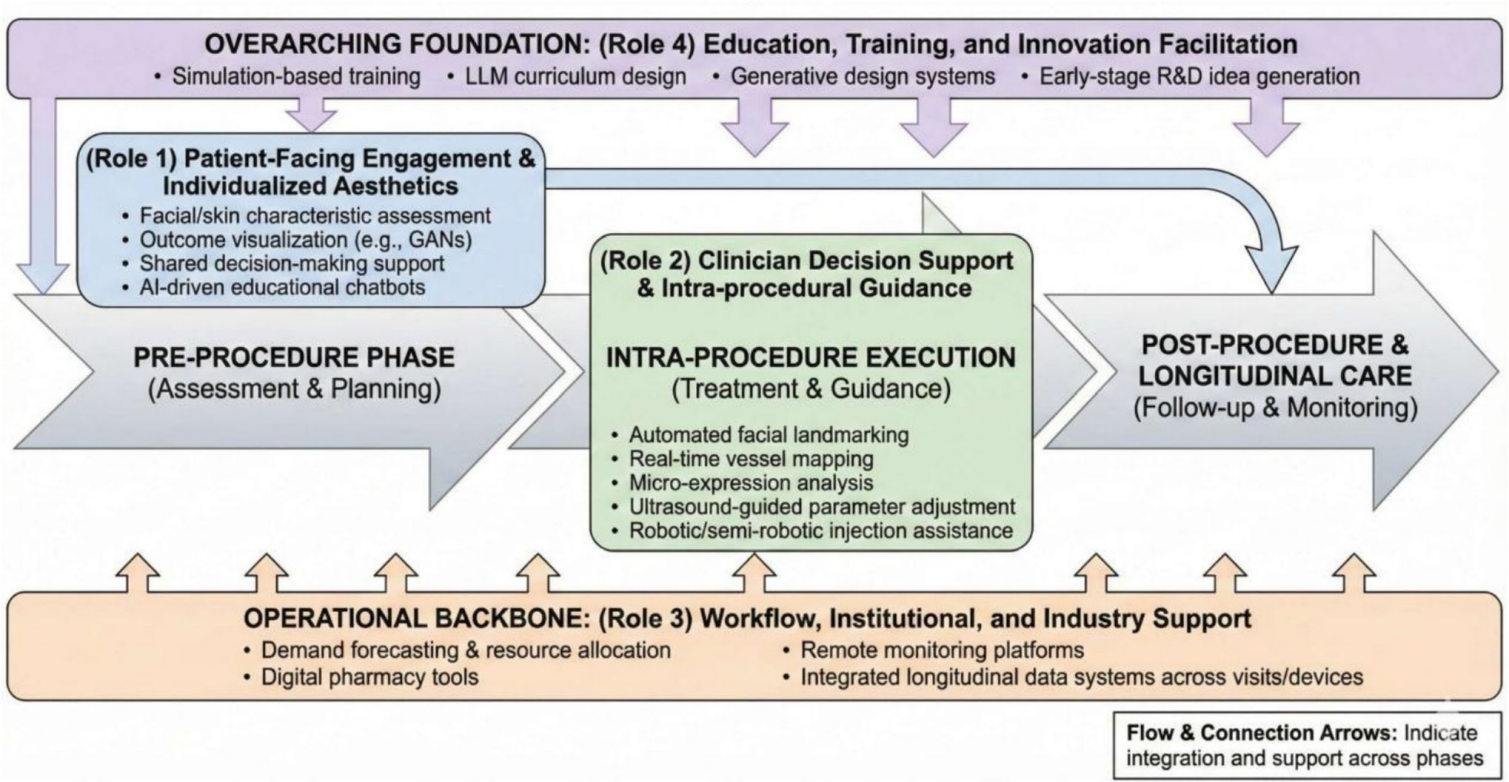
Functional taxonomy & workflow integration of Al in non-surgical cosmetic

The 89 included studies describe a broad spectrum of AI applications across the non-surgical cosmetic workflow. To avoid redundancy and to clarify the distinct contributions of different technologies, we organize the findings into a functional taxonomy comprising four primary roles (Fig. 2). The first role, patient-facing engagement and individualized aesthetics, includes tools that directly interact with patients to assess facial or skin characteristics, visualize potential outcomes, provide educational content about procedures, and support shared decision-making, such as facial attractiveness estimators, skin analysis applications, and AI-driven chatbots. The second role, clinician decision support and intra-procedural guidance, consists of systems that augment physician assessment, planning, and execution, including automated facial landmarking, vessel mapping, micro-expression analysis, ultrasound-guided parameter adjustment, and robotic or semi-robotic injection assistance. The third role, workflow, institutional, and industry support, involves AI applications designed to improve operational efficiency and strategic decision-making, such as demand forecasting, digital pharmacy tools, remote monitoring platforms, and integrated data systems supporting longitudinal care. The fourth role, education, training, and innovation facilitation, encompasses platforms using AI—such as large language models and generative imaging systems—to support clinician education, simulation-based training, curriculum design, and early-stage idea generation for research and product development. Subsequent subsections elaborate these roles. Where AI contributions to personalization, safety, and engagement overlap across roles, we avoid redundancy by referring back to this taxonomy. For example, patient-centric initiatives and individualized aesthetics fall primarily under role one, whereas safety-oriented image guidance and complication mitigation belong mainly under role two.

### Study Outcome Meet Cosmetic Need

#### Define Individualized Aesthetics

**Fig. 3:**
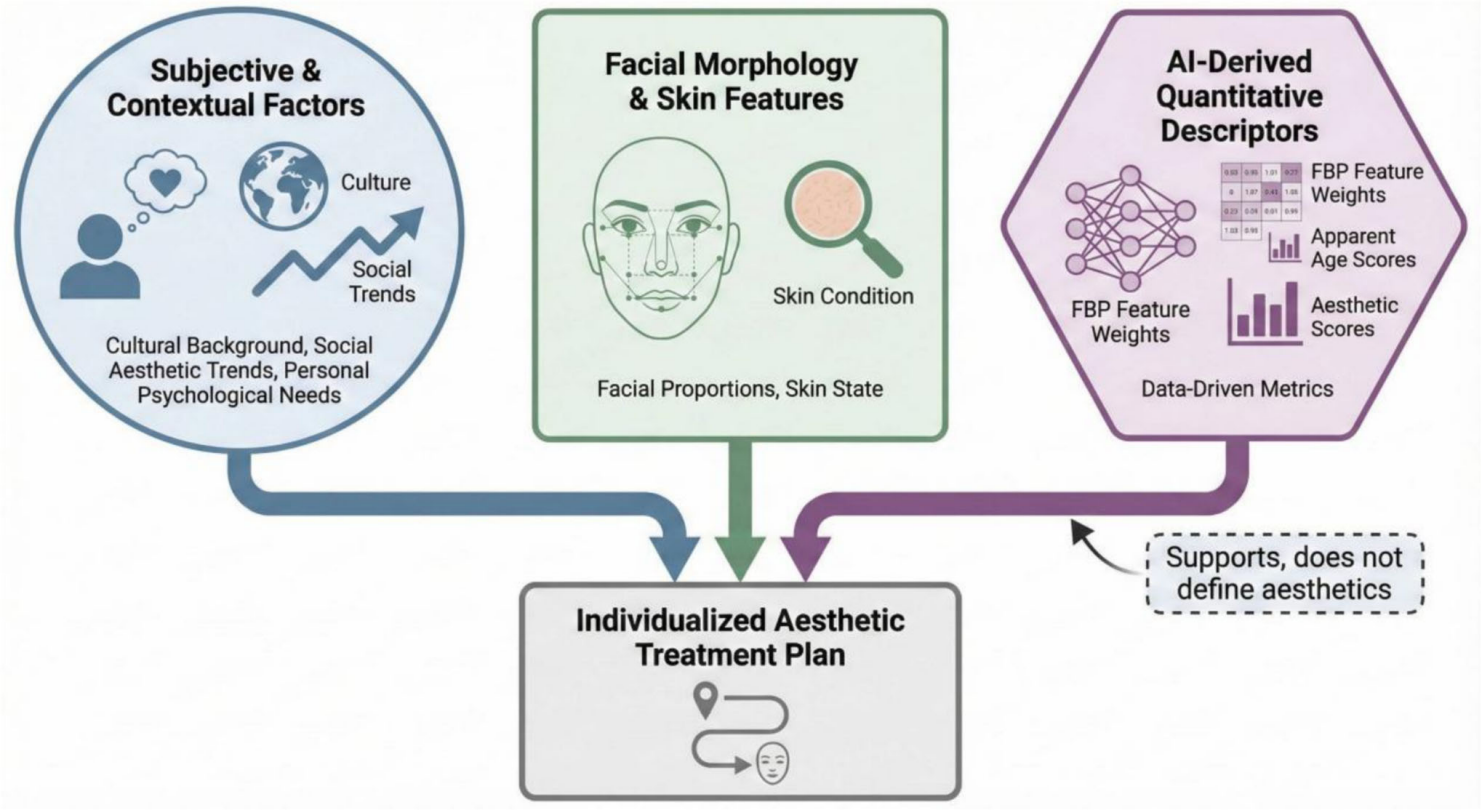
AI to define individualized aesthetics

Individualized aesthetic emphasizes the uniqueness and differentiation of individual facial features, and shaped by cultural backgrounds, social aesthetic trends and individual psychological needs [8–10]. And precise assessment combining subjective preferences with objective data needs to be achieved through technological means (e.g., AI) [6, 11]. Central to this transformation is AI’s ability to analyze multifaceted patient data, from facial morphology and skin characteristics to lifestyle preferences, enabling tailored treatment strategies that align with individualized aesthetic goals (Fig. 3).

Combining global and local interpretability techniques, facial beauty prediction (FBP) models reveal how AI models can quantify the contribution of facial features to attractiveness: aesthetics relies on multi-feature synergy—interaction of eyes, nose, cheeks, while chin and lips are less important than expected; Asian dataset features influence model preferences—focus more on nasal structure [12]. This is very similar to realistic global physician survey [13]. Takanori has a similar study result but also extends the psychological findings by evaluating impressions [14].

Conversely, the Python-based algorithm was used to study frontal patterns and see what effect they have on beauty standards in public images of female celebrities from around the world. Irrespective of race background, an appealing female face is characterized by a zygomatic-to-mandibular width ratio nearing 80%, a mid-facial third that is slightly larger than the lower third, and a distinctive chin angle of approximately 138 degrees, contributing to a trapezoidal facial shape [15].

To draw parallels between the evaluation of facial attractiveness of adult female white faces ascribed by human and AI-based websites, a strong positive and linear correlation was observed between the AI and expert human scores. This correlation was found to be independent of the gender and race of the human experts [16].

As it is proved, facial attractiveness can be calculated using face detection, deep convolutional neural networks, support vector regression for facial beauty, and collaborative filtering technology. Several factors (e.g., expression, race, and ethnicities) that interfere with judgment need to be included to train the model. These are also aligned with treatment outcome evaluation principal [17, 18].

Furthermore, initially focusing on objective beauty criteria (feature-based aesthetics), later studies suggested that beauty results from the interaction between the object and the perceiver [19]. Nicolas’s deep convolutional neural networks (DCNN) analysis revealed that neural sparsity accounts for 28% of aesthetic variance (47% with feature metrics), quantifying the impact of brain processing efficiency on aesthetics [20].

#### Customize Facial Assessment

**Fig. 4:**
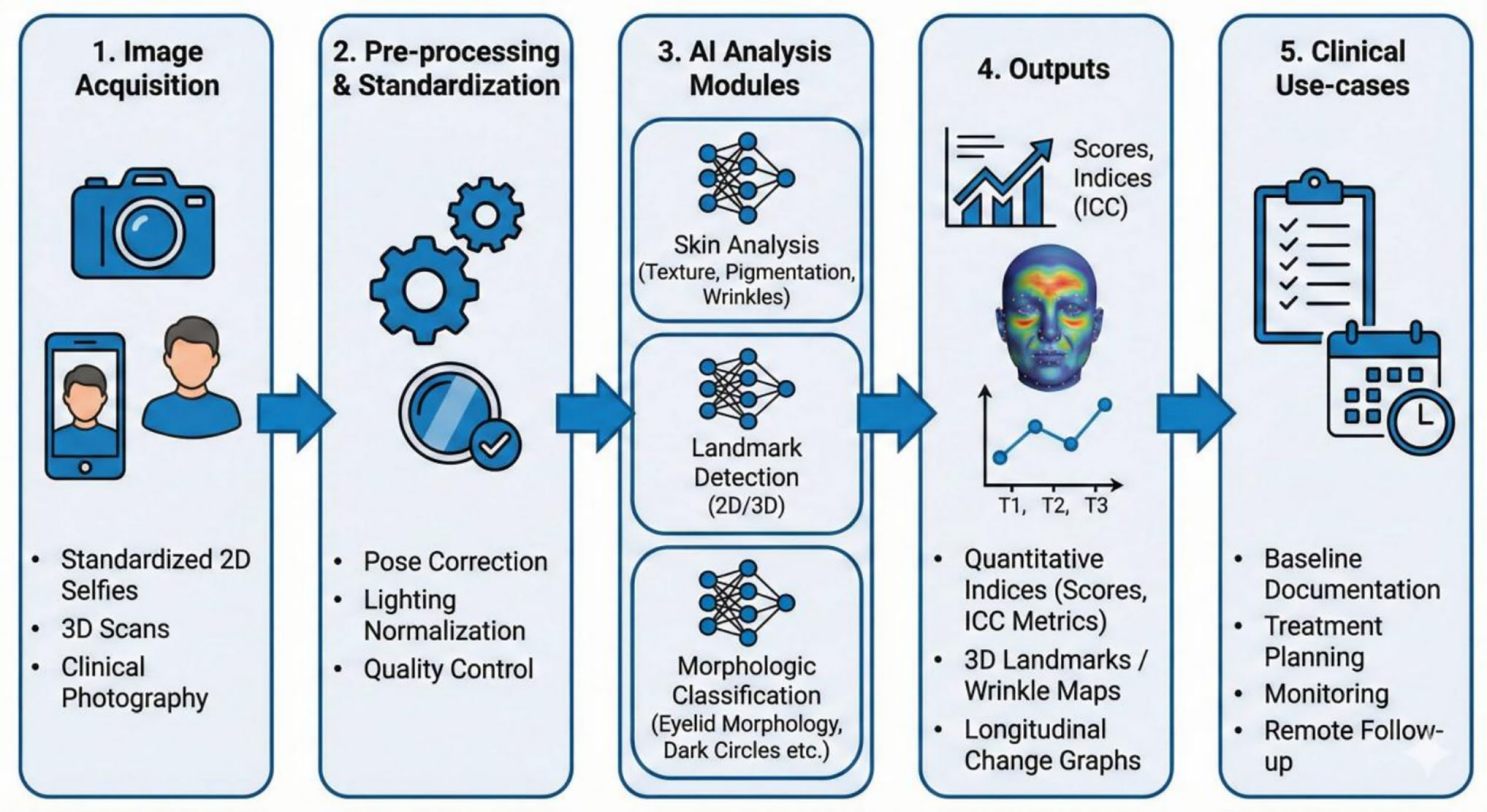
AI to customize facial assessment

Historically, facial assessment relied on manual measurements by physicians or basic 2D digital imaging [21, 22]. Advances in photographic standardization have since improved imaging accuracy [23], enabling AI-driven tools to transform facial assessment. AI applications in this domain focus on two core capabilities: automated facial feature classification and precise landmark detection (Fig. 4).

For instance, the EfficientNet-V2M model leverages diverse datasets and optimized architectures to deliver multi-dimensional skin analysis (e.g., Fitzpatrick texture, hyperpigmentation, redness, and wrinkles) from facial photographs, achieving an average accuracy of 85.41% [24]. Deep learning algorithms further enable objective evaluation of eyelid morphology, quantifying pre- and post-intervention changes [25, 26]. Smartphone-based AI tools analyze 2D selfies to assess dark circle severity and correlate findings with seasonal skin texture variations and lifestyle factors, demonstrating AI’s capacity for customized ocular assessments [27]. Nonetheless, a comprehensive facial feature classification should encompass numerous parameters, including but not limited to the following: the facial golden ratio, symmetry, proportion, side angles, skin condition, overall harmony, personality, temperament, and social attraction [28].

Specialized platforms employing patented scales or wrinkle segmentation algorithms outperform manual raters in consistency and timeliness, facilitating remote monitoring and real-time wrinkle tracking [29, 30]. Advanced 3D imaging systems automatically identify 21 anatomical landmarks on soft tissue scans with inter-/intra-rater reliability exceeding ICC > 0.9 and a mean deviation of 3.2 mm compared to manual annotations [31]. Additionally, smartphone applications integrating facial landmark detection technology (e.g., FAIN system) ensure dynamic calibration during imaging, enhancing consistency for aesthetic monitoring [32].

#### Tailored Patient Education

Patients are increasingly adopting a rational decision-making approach, prioritizing value-for-money and premium-quality services. Predictable outcomes and testimonials from peers/family exert primary influences on treatment choices, while social media and celebrity endorsements play a negligible role [3].

Contemporary patient assessment begins at the initial reception encounter. A comprehensive aesthetic consultation should integrate thorough medical history-taking, risk stratification, and anatomical aging education. Clinicians must guide patients to adopt a holistic facial evaluation paradigm, leveraging pre-/post-procedure photography to contextualize treatment goals—a critical strategy given patients’ propensity to focus on isolated features rather than global aesthetics [10]. However, suboptimal consultation durations often constrain these efforts, and AI is a potential solution to the issue.

Large language models (LLMs) demonstrate certain performance in addressing patient inquiries, covering topics such as procedural indications, safety profiles, recovery protocols, and cost considerations. Expert evaluation confirms their potential as ancillary educational tools [33]. For instance, ChatGPT provides accurate information on botulinum toxin (77.1%) and dermal fillers (62.9%), with over 65% of physician-reviewed content deemed medically sound [34]. Nevertheless, its responses exhibit low inter-rater reliability (ICC≈0) and struggle to capture nuanced procedural details. While LLMs emphasize personalized approaches in aesthetic surgery, their utility remains limited in complex counseling scenarios [35]. After all, machine learning capability is a key factor in improving performance as a patient counselor or education assistant.

Advancements in AI-driven interactive tools support postoperative care. Chatbots can deliver real-time guidance, address concerns, and reinforce medical directives in alignment with clinical guidelines [36]. Notably, AI-powered skin aging simulations significantly enhance young women’s awareness of UV protection and motivate preventive behaviors [37]. Shi et al.’s “Skincare Mirror” system exemplifies this trend, using predictive visualization to engage users—particularly males—in skincare adherence [38]. Such technologies democratize access to specialized knowledge, particularly benefiting underserved populations, and optimize long-term aesthetic outcomes through sustained patient education.

#### Predict Achievable Outcome

Cosmetic procedures demand exceptional precision due to the anatomical complexity and individual variability of the human face. To meet these demands, AI-driven predictive modeling has the potential to be an important tool of treatment planning. For instance, the AutoGluon AutoML framework achieved an 82–88% accuracy rate in simulating rhinoplasty outcomes by integrating preoperative morphometrics with consensus aesthetic scoring [39].

Advanced metric models further enhance prognostic precision. The CAARISMA® ARMM platform synergizes the Facial Youth Index (FYI), Aesthetic Index (FAI), and Skin Quality Index (SQI) to deliver objective postoperative evaluations in facial transplantation, achieving a 0.89 intraclass correlation coefficient for aesthetic improvement validation [40].

Innovative CNN-based platforms extend predictive capabilities to age-related outcomes. Studies utilizing FacePlusPlus (China) and Amazon Rekognition (USA) demonstrated that convolutional neural networks predicted a preoperative age reduction of 2.4 years versus chronological age, escalating to a 3.2-year reduction post-blepharoplasty. Notably, patients presenting with advanced chronological aging exhibited the most pronounced perceptual age reversal [41].

Augmented reality (AR) and virtual reality (VR) technologies can change preoperative visualization. The Crisalix VR 4D platform, for example, constructs 4D facial models incorporating dynamic tissue deformation parameters, enabling real-time simulation of rhinoplasty, rhytidectomy, and lip augmentation outcomes [23, 42]. ModiFace’s proprietary AR system has been shown to reduce decisional regret by 43% in cosmetic consultations by enabling immersive preview of treatment results [43].

#### Support Full-Scope Assessment

State-of-the-art 3D imaging technology can accurately assess the basic conditions of the patient before surgery, and can more intuitively restore the anatomical structure of the surgical site, and through the adjustment of the system parameters can simulate the different surgical effects of different surgical procedures and different access diameters, so as to carry out a more perfect surgical program design, and to effectively avoid unnecessary injuries in the course of the surgery. 3D imaging technologies (such as laser scanning, structured light scanning, and stereoscopic photography) are being developed to provide further volume and side recognition of the face. Each of these three technologies has its own characteristics and limitations in terms of cost, portability, accuracy, and application scenarios [44]. The DeepBeauty3D algorithm exemplifies this capability, automatically extracts anatomical features from CT images, and completes the aesthetic scoring of bones and soft tissues in 11.2 seconds (with an error rate of 4.62% and 2.00%, respectively) [45].

Ultrasound imaging optimization is a key consideration to address clinical requirements and challenges. Tailored AI plans are capable to enhance diagnostic accuracy by addressing the unique complexities and variability inherent in ultrasound examinations [46]. 3D Line-field Confocal Optical Coherence Tomography (LC-OCT) imaging coupled with AI-based quantification algorithms reveal the patterns of epidermal thickness, keratinocyte network density, and nuclear volume changes with age, which provide new quantitative biomarkers for facial skin aging, especially in the temporal, zygomatic, and mandibular regions [47, 48]. Ultrasound imaging of facial vascular neural structures has been emphasized to the safe aesthetic injections recently [49]. DeepRAP uses deep learning to automatically divide ultra-wideband raster-scan optoacoustic microscopy (RSOM) images into 3D and calculate various skin features and blood vessel structures under the skin [50]. The truth like aging affects small vessels in the upper dermis is confirmed accordingly.

Furthermore, in treatments involving radiofrequency or ultrasound-based skin tightening, the AI system adjusted the intensity and duration of energy delivery in real time, based on feedback from ultrasound imaging [46, 51]. And computer vision technology enhances cosmetic procedures by providing precise facial analysis for optimal injection sites, ensuring natural results and patient safety [52].

#### Optimize Effective Treatment

In the pursuit of a natural and balanced treatment outcome, it is imperative to consider the emotional and facial expressions exhibited by patients [22]. AI-based Customized Precision Facial Assessment (CPFA) quantifies both static and dynamic micro-expressions by detecting facial action units, enabling targeted interventions (e.g., neuromodulators and fillers) that address specific aesthetic concerns [53–55]. Otherwise, five quantitative smile assessment parameters used as reliable predictors of smile simulation can also support this precise treatment [56].

AI-powered tools provide real-time guidance during filler injections by overlaying digital markers on live video feeds, indicating precise injection points and depths. This technology improves injection accuracy and consistency while reducing complication risks [57].

An intelligent evaluation process based on convolutional neural networks (CNNs)—encompassing data collection, enhancement, model training, and final efficacy grading—enhances objectivity and standardization in non-invasive photoelectric therapy [58]. The Kesty AI model demonstrates that combining CO_2_ laser blepharoplasty for upper eyelids with Er:YAG laser resurfacing for lower eyelids effectively achieves periorbital rejuvenation [59].

Deep learning integration facilitates personalized skincare regimens tailored to individual genetic and environmental profiles. SNP analysis proves valuable for studying predispositions to skin features like collagen degradation, pigmentation, and inflammatory responses. Combining AI with enhanced SNP profiling and epigenetic insights enables more accurate predictions of individual treatment responses [60].

Robotic systems like NextMotion are used for precise filler and toxin injections, featuring advanced imaging, control systems, and sensory feedback [61]. Similarly, ARTAS systems perform follicular unit extraction for hair restoration, while robot-assisted laser hair removal (LHR) systems ensure uniform laser distribution with fewer side effects [62, 63]. However, current robotic solutions remain expensive, inflexible, and potentially hazardous if malfunctions occur.

**Fig. 5:**
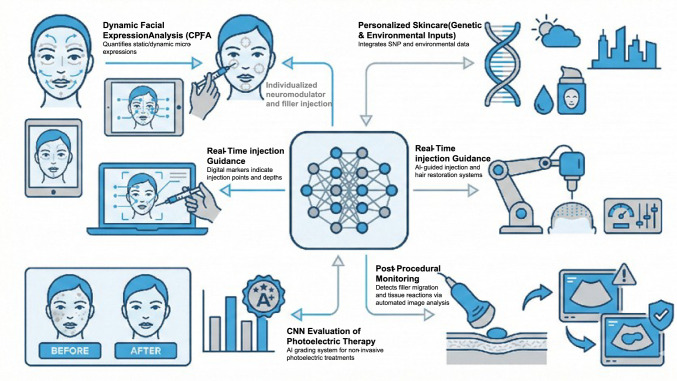
AI to optimize effective treatment

Machine learning-based binary recursive partitioning successfully identifies unknown complication sources and determines key feature relationships [64]. A multichannel CNN-based eye model significantly improves surgical repair outcomes in ophthalmic plastic surgery, reducing postoperative complication rates from 28 to 13% (*P*<0.05) compared to physician-only methods [65]. AI also enables automated analysis of post-procedure ultrasound images, comparing them with baselines and expected outcomes to quickly detect issues like filler migration or abnormal tissue reactions, allowing immediate corrective action (Fig. 5) [66].

#### Facilitate Influence Spread

Internet technology’s impact on cosmetic surgery is evident through significant field advancements. The 50 most disseminated online articles had a mean Altimetric Attention Score (AAS) of 11.3, primarily driven by Twitter mentions, with no correlation between AAS and citation count. Plastic and reconstructive surgery published 26% of these articles, receiving higher AAS than other journals. Analysis revealed substantial collaboration in AI-related plastic surgery research: 64% of articles were multi-institutional and 34% multinational, though only 16% had female first authors, suggesting potential gender bias in the field [67].

The influence of social networks, photo-editing applications, and artificial intelligence on cosmetic surgery patients warrants further investigation. A study found that 107 patients chose their surgeons based on social media presence rather than professional reputation or website content [68]. Medical practitioners may employ ChatGPT to enhance their marketing efforts by generating customized educational content for prospective patients. The model can produce various marketing materials, including blog posts and social media content, which effectively communicate procedure details, risks, benefits, and recovery information. By providing valuable information to potential patients, physicians can establish their expertise and attract a larger patient base [69].

As cosmetic products become increasingly homogeneous, physicians maintain critical roles in product R&D by providing clinical insights to manufacturers. They contribute to product positioning, indication expansion, and application technology development [70]. ChatGPT shows capability as a tool for discovering novel aspects of cosmetic surgery and identifying innovation opportunities. Evaluation of ChatGPT responses to plastic surgery inquiries demonstrated its ability to generate relevant, accurate, and novel insights [71]. When prompted to develop rhinoplasty patents, ChatGPT achieved 45% predictive power [72]. For generating systematic review ideas in cosmetic surgery, ChatGPT showed 35% accuracy for general concepts and 75% for specific topics [69].

#### Advance Medical Education

Cosmetic procedures are characterized by their specialized nature, broad scope, and rapidly evolving knowledge base. These procedures intersect with multiple disciplines and exhibit significant patient-specific variability, necessitating comprehensive expertise among practitioners. However, current training methods remain limited by condensed curricula and unidimensional approaches [73].

AI demonstrates potential to improve training quality and efficiency. ChatGPT’s performance on the USMLE Step 3 improved from 56.9% accuracy (version 3.5) to 84.7% accuracy (version 4.0) [74]. A comparative study of seven AI platforms on the 2019-2023 Plastic Surgery In-service Training Examination (PSITE) confirmed ChatGPT-4.0’s effectiveness as a plastic surgery educational tool [75].

Two primary avenues exist for educational enhancement: (1) simulation-based training and (2) artificial intelligence integration. Thomson classified 12 existing simulators into four categories: computer-based (5), synthetic (2), animal (2), and cadaver (3). AI-powered simulators enable trainees to practice surgical techniques in safe, controlled environments, reducing errors and complications during actual procedures. Machine learning algorithms analyze trainee performance videos to assess skill proficiency, identify improvement areas, and predict surgical outcomes, functioning as real-time self-assessment tools [76]. The DALL·E 2 text-to-image system generated clinically accurate images for medical education, with particular success in depicting soft tissue tumors and less accuracy for wound representations [77]. Virtual patient learning systems employing intelligent robotics allow repeated practice of clinical skills, enhancing film-reading abilities and procedural mastery [78].

Finally, AI can generate comprehensive curricula for specific procedures like facial botulinum toxin injections. These AI-designed programs provide detailed learning objectives, structured two-day schedules, and hands-on practice resources, demonstrating scalability for large-scale aesthetic medical education initiatives [79].

#### Support Institutional/Industrial Operation

The integration of artificial intelligence (AI) and machine learning into healthcare offers significant potential to optimize pharmacy operations. These technologies enable continuous patient monitoring and personalized treatment adjustments, thereby improving clinical outcomes. Key benefits include:

- Enhanced drug development and clinical trial optimization
- Identification of medication usage patterns
- Detection of potential drug interactions
- Reduction of prescription errors

Additionally, machine learning provides more efficient alternatives to traditional high-throughput screening (HTS) methods [80].

**Fig. 6:**
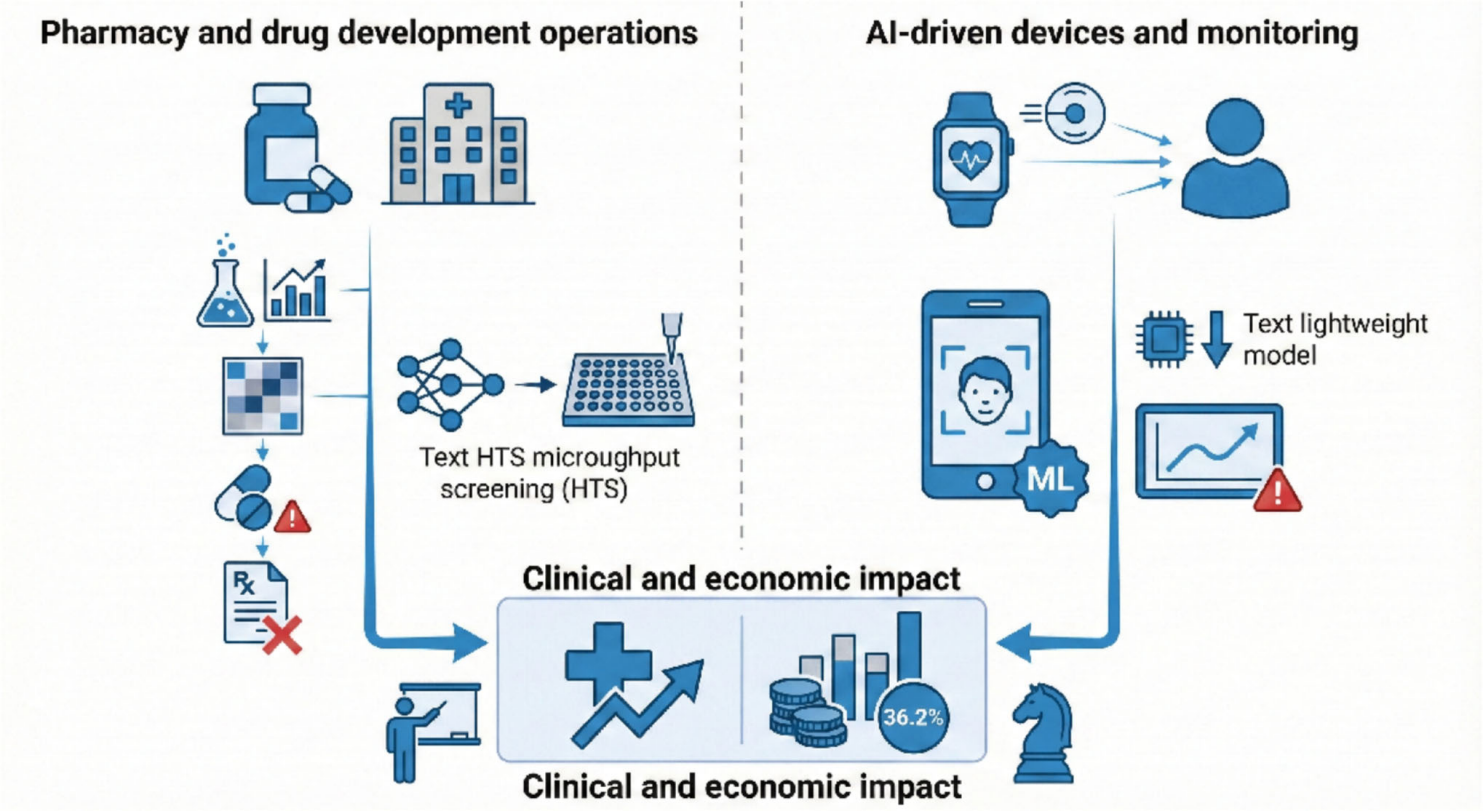
AI to support institutional/industrial operation

AI-driven devices show promise as clinical differentiators. AI algorithms can monitor patient progress by analyzing data from wearable devices or medical sensors. Lightweight models developed for low-resource settings can be deployed on-site without requiring patient data sharing, cloud upload, or high computational power—with some systems adaptable to smartphone platforms. For example, Facekit is an Android smartphone application for rapid facial feature analysis that utilizes ML Kit, Google’s open-access computer vision Application Programing Interface (API) developed by Google [81–83]. These real-time monitoring systems facilitate early complication detection, prompt interventions, and personalized postoperative care. However, a recent survey revealed that only 36.2% of respondents reported positive financial outcomes from AI implementation, underscoring the need for targeted training and strategic approaches to maximize AI’s clinical and economic impact (Fig. 6) [84].

### Factors Limit AI’s Application

#### Limited or Biased Datasets

Artificial intelligence systems demonstrate significant dependence on training datasets, where data quality directly impacts output reliability. Insufficient or unbalanced datasets can compromise learning efficiency and result in inaccurate predictions. Notably, studies have identified inherent biases within AI models, including irrational prejudices and discriminatory patterns [85]. This issue is particularly evident in generative adversarial network (GAN) variants, which have been shown to amplify gender and skin tone biases in generated data [86].

The field is witnessing rapid advancements in automated facial landmark detection and analysis, marking the emergence of measurement-based automatic recognition systems. However, the limited availability of 3D imaging devices currently restricts these applications. To address this gap, researchers should prioritize constructing comprehensive 3D facial databases using existing 3D body surface imaging technology. Critical considerations during database development include:

- Ensuring data privacy protections
- Maintaining dataset comprehensiveness
- Addressing potential biases in data collection

The integration of automatic marker recognition technology enables the fusion of 3D facial features with multimodal imaging data (CT, MRI, etc.), facilitating the development of more accurate recognition models [44].

Empirical evidence from OpenAI’s ChatGPT evaluation across six national medical licensing exams reveals significant performance variations. While achieving 73% accuracy on Italian examinations, the system only attained 22% accuracy for French examinations, with particular deficiencies in questions requiring multiple precise responses [87]. This underscores the need for expanded cross-national research to better understand geographic and linguistic influences on AI performance.

#### Unreliable Algorithms

Fundamentally, most current AI algorithms remain grounded in supervised learning frameworks, which inherently constrain their reliability in healthcare settings. Three critical constraints emerge: (1) extensive high-quality training data requirements for each technical skill acquisition, (2) susceptibility to overfitting in medical scenarios where models excessively adapt to training data, and (3) algorithmic complexity that obscures interpretability due to numerous variables. These factors collectively limit AI system performance, where effectiveness depends not only on algorithmic sophistication but also on access to substantial, high-quality datasets [88].

ChatGPT, for instance, has demonstrated a propensity to produce responses that appear reasonable yet often contain inaccuracies or lack coherence. This was substantiated in 27 documented cases where ChatGPT proposed systematic review topics for which no existing published literature could be identified through MeSH term searches [69].

Notably, significant omissions were found in ChatGPT’s discussion of breast augmentation risks, including suboptimal word choices that could mislead patients and an unclear ability to contextualize statements based on varying evidence levels—factors that may adversely affect informed consent and patient expectations [33]. In postoperative care guidance for facelift and neck lift patients, ChatGPT achieved 85% accuracy in differential diagnosis but only 56% accuracy in recommending appropriate patient dispositions. Its tendency to overestimate urgency and include excessive information could potentially increase healthcare resource utilization and patient anxiety [89].

Digital deprescribing tools show promise in personalizing medication reduction through interactive AI features, yet consistently neglect integrated assessment of psychological needs and health literacy disparities across populations [90]. Evaluation of four major LLMs in abdominoplasty patient education revealed marked performance divergence: Claude’s protocols proved most feasible, while Copilot achieved highest patient-friendliness scores. However, significant variability emerged across readability, professionalism, and utility metrics, necessitating strict scenario-specific selection. Researchers advocate for incorporating real patient data and expanding professional review panels to enhance external validity [91].

#### Physician/Patient Trust Deficit

A 2024 IMCAS Congress survey showed 91.5% of participants possessed substantial AI knowledge in cosmetic medicine, yet only 48.9% had implemented AI tools in practice. This adoption rate mirrors Vivacy’s 2023 data but with heightened concerns (50%) about AI displacing human labor [84]. In comparison, a 2019 study found merely 5.5% of respondents agreed dermatologists would be replaced by AI in the foreseeable future [92]. A 2024 Latin American survey revealed 82.1% of respondents reported limited/no experience with AI in plastic surgery [93]. These variations suggest adoption is influenced by multiple factors including geographic location, professional specialty, and temporal trends in technological acceptance.

Indeed, there truly is doubt in current AI. For example, achieving objectivity in AI-driven aesthetic assessments remains challenging due to the inherent subjectivity in scoring. If the scores given by the raters in the training data are not objective, then the scores predicted by the model will not be objective either. To obtain more objective scores, research attempt to mitigate by aggregating multiple opinions to establish a consensus score [39]. While 3D/4D imaging integration shows promise for outcome visualization, smartphone-based assessments risk distorting patient perceptions. Cloud storage systems offer operational advantages but introduce data breach vulnerabilities [23].

While recognizing AI’s potential to enhance healthcare delivery, stakeholders emphasize preserving human-centered care; and citizen engagement strategies should prioritize AI literacy programs to foster informed participation in governance [94].

## Discussion

### Principal Findings

This review on AI applications in non-surgical cosmetic medicine and organized them into a functional framework spanning patient-facing tools, clinician decision support, workflow and institutional solutions, and education and innovation facilitation. Across this framework, AI is being used to translate subjective aesthetic judgments into more standardized measurements, to assist with imaging and anatomical interpretation, and to support remote monitoring and training.

While pursuing cost-effective cosmetic, patients are more in pursuit of quality cosmetic services and treatment experiences that are both safe and personalized. especially favoring the continued consumption of photoelectric and injectable projects [3, 95]. The integration of AI in cosmetic procedures facilitate patient-centric care by addressing the evolving demands of individuals seeking personalized, efficient, and safe aesthetic solutions [96]. In the realm of injectable treatments, AI-driven platforms leverage deep learning algorithms to assess facial asymmetry, skin elasticity, fat distribution, and even muscle movement [46, 55, 97, 98]. These systems also prioritize patient safety by identifying contraindications, such as proximity to blood vessels, thereby reducing risks like bruising or blindness. Photoelectric and skin care could be redefined through AI-powered skin analysis [32, 58]. AI-driven apps like SkinAnalysis generate hyper-personalized skincare regimens by correlating phenotypic traits (e.g., Fitzpatrick skin type) with environmental factors (e.g., UV exposure) [24]. Such platforms empower patients to make informed decisions about laser treatments, chemical peels, and product usage, enhancing treatment compliance and outcomes.

As cosmetic procedures escalate, clinicians face mounting demands for precision, efficiency, and adaptability. In addition to the evaluation of facial features, the identification of the appropriate treatment area, and the calculation of product quantities, these processes have the potential to be subjective and time-consuming. Artificial intelligence emerges as a vital copilot in this landscape, addressing key pain points by providing multi-dimensional decision support throughout the procedural workflow [99].

The digital pharmacy market is projected to grow at a 14.42% annual rate, reaching $35.33 billion by 2026, while McKinsey estimates AI could generate nearly $100 billion annually in the U.S. pharmaceutical sector [80]. While current AI decision-support systems showed modest cost-effectiveness improvements ($750 vs. $759 per quality-adjusted life-year), future advancements may enhance their economic value. Further research into regulatory frameworks, adoption incentives, and diagnostic process efficiency is needed to clarify AI’s cost-effectiveness, particularly in non-specialist patient screening roles that improve referral targeting [100].

To advance understanding of consumer interactions with emerging technologies, six key research areas have been identified: rethinking consumer behavior models, generational differences in technology adoption, consumer interactions with automated services, ethics, privacy, and algorithmic transparency (“black box” issues), consumer security concerns, technology adoption during and after global crises [101].

However, most applications are still at an early developmental stage: the literature is dominated by technical or proof-of-concept studies with limited clinical validation, and outcome measures frequently emphasize algorithmic performance or user satisfaction rather than hard clinical endpoints. Taken together, the current evidence supports viewing AI as an adjunct that can enrich and structure aesthetic practice, rather than as a mature technology ready for autonomous or routine deployment.

### Quality of Evidence and Clinical Implications

The overall quality of evidence supporting AI in non-surgical cosmetic procedures is low. The majority of studies are proof of concept or technique validation, employ single-center datasets, and internal validation only, with small to moderate sample sizes relative to model complexity. Reporting of dataset construction and demographic composition is often incomplete, restricting the ability to assess representativeness and subgroup performance. Prospective evaluations and randomized or controlled comparisons with expert clinicians are scarce, and very few studies examine clinically meaningful outcomes such as complication rates, functional improvement, patient-reported satisfaction, or cost-effectiveness [102].

Given these constraints, AI tools in non-surgical cosmetic practice should be positioned as structured aids to assessment and communication rather than as replacements for clinical judgment [88]. For example, patient-facing models can help convert diffuse aesthetic impressions into reproducible metrics and facilitate alignment of expectations, but they must be interpreted within the context of individual goals and cultural norms, and they should not be treated as definitive arbiters of beauty or rejuvenation [103]. For clinicians, imaging-based AI systems and decision-support tools have potential to enhance standardization, documentation, and safety planning, particularly around complex anatomical regions. However, the lack of robust comparative data means that their incremental benefit over experienced practice is uncertain, and integration into busy workflows may require substantial time, training, and infrastructure [97]. Institutions considering adoption of AI for workflow optimization, remote follow-up, or automated patient communication must balance anticipated gains in efficiency and consistency against costs, implementation challenges, and the risk of overreliance on systems whose reliability has not been fully established. Large language models and automated content-generation tools may assist with education and communication, but their use raises additional questions about information accuracy, bias, and professional responsibility that require explicit governance [34].

### Suggestion to Alleviate AI’s Limitation

Challenges associated with integrating AI into cosmetic procedure include concerns about data and its privacy, algorithmic bias, and the need for comprehensive training of physicians in the effective use of AI-driven technologies. Collaboration between clinicians, technologists, and regulators is critical to developing robust AI solutions that are ethical and prioritize patient safety.

In order to deal with limited or biased datasets, future research directions should focus on developing robust, reliable databases, creating small-sample recognition systems for individualized needs, ensuring patient safety through high-quality training data and enhancing model performance via multi-institutional data retraining.

To optimize ChatGPT’s utility, researchers recommend strategies such as providing clear prompts, incorporating examples, using interactive refinements, and verifying information through cross-referencing with trusted sources [104]. For multi-model applications, evidence suggests combining complementary LLM strengths could improve educational outcomes. Future research should investigate how LLM-generated advice impacts patient outcomes and satisfaction, while also exploring methods to enhance response accuracy, detail, and personalization in medical contexts [91].

Multiple surveys have examined stakeholder perspectives on AI implementation in healthcare. While knowledge levels appear high, actual adoption rates remain modest, revealing a persistent trust gap. Bridging the trust gap requires targeted educational initiatives. 70.2% of survey respondents identified critical needs for expanded AI education and institutional support [84]. Enhanced practitioner understanding directly correlates with improved clinical integration and risk mitigation [5]. Secondly, incorporating AI fundamentals into medical education prepares future professionals to leverage technology while recognizing its limitations [93]. Finally, effective solutions demand interdisciplinary partnerships where developers possess domain-specific clinical knowledge [5].

### Regulatory and Ethical Considerations

AI applications in facial surgery require processing large volumes of facial images containing identifiable personal data. Beyond storing mere images, these systems now extract and analyze biometric information that often exceeds the original visual content. This creates significant consent challenges, as conventional mechanisms struggle to ensure patient privacy when dealing with highly recognizable facial data [105, 106].

To address these concerns, structured evaluation frameworks have emerged. A specialized checklist guides dermatology AI algorithm assessment, covering dataset curation, model development, and performance evaluation while highlighting ethical risks and bias mitigation strategies [107]. In addition, compliance with healthcare privacy regulations (e.g., HIPAA) remains essential for protecting sensitive patient information. Patients must receive comprehensive disclosures about AI’s role, including data usage protocols and potential risks, with documented informed consent [91].

Secondly, emerging risks require multifaceted safeguards. For example, ChatGPT can generate convincing fake patient reviews, deceiving both human evaluators and commercial AI detectors, highlighting the need for strategies to differentiate real from AI-generated content. Analyzing emotional tone and review length offers preliminary tools for authenticity detection, but comprehensive solutions require enhanced digital literacy among patients and physicians to combat misinformation ecosystems [108]. As a result, maintaining human oversight remains paramount to preserve medical judgment quality and mitigate AI-related risks [91].

The development of AI technologies faces unique validation challenges that may limit real-world applicability. The SPIRIT-AI and CONSORT-AI guidelines, which establish standardized reporting protocols for clinical trials involving AI interventions in medicine and dermatology [109, 110].

But debates about AI’s civil subject status reveal three perspectives:

1. *Affirmative Position* Strong AI should bear legal responsibility for medical malpractice as an independent entity.
2. *Negative Position* Current weak AI systems cannot assume legal liability due to inherent technical limitations.
3. *Compromise Approach* Proposes graduated responsibility allocation based on:
  - Degree of AI intelligence
  - Physician oversight levels
  - Scope of AI autonomy

This framework advocates shared liability across the entire R&D-application continuum [111, 112].

### Limitations of this Review

Several methodological limitations of this review should be acknowledged. The search was limited to studies published in English or Chinese within a defined time window, and other relevant work may have been missed. Gray literature, preprints, and patents were not systematically searched, introducing potential publication bias. Marked heterogeneity in study design, AI architectures, datasets, endpoints, and comparators precluded quantitative meta-analysis, so the synthesis is necessarily narrative and thematic. No single risk-of-bias tool was applicable across all included designs; instead, a domain-based appraisal was developed and applied by two independent reviewers with adjudication of disagreements, which introduces an element of subjectivity. Incomplete reporting of demographic characteristics, dataset construction, and funding sources in the primary studies further constrained detailed analysis of fairness, subgroup performance, and conflicts of interest. Finally, AI in healthcare is evolving rapidly, and newer systems and evaluations may not be captured by the present review.

## Conclusion

Artificial intelligence is increasingly being integrated into non-surgical cosmetic practice and appears capable of enhancing precision, personalization, and efficiency across pre-, intra-, and post-procedural stages. By leveraging computer vision, predictive modeling, and large language models, current systems can structure aesthetic assessment, enrich patient counseling, and support procedural planning and monitoring. These developments coincide with the rapid growth of the global non-surgical aesthetic market, particularly in Asia, and align with the field’s broader shift toward technological diversification and changing consumer demographics. At the same time, the available evidence remains limited in scope and quality, and most applications are still in early phases of clinical translation.

Within this emerging landscape, AI has the potential to support different stakeholders in complementary ways. To be patient-centric initiative, patients may benefit from more transparent and individualized assessments and from enhanced access to structured educational resources. To be physician copilot, clinicians can draw on AI-assisted imaging, risk stratification, and simulation tools as adjuncts to their own judgment, as well as on AI-enabled platforms for continuous professional education. For Institutional/industrial development, institutions and industry actors may use AI-based analytics to optimize workflows and to identify opportunities for innovation. However, these benefits are contingent on the careful design, validation, and governance of AI systems; they cannot be assumed from technical feasibility alone.

Despite progress, critical limitations persist. Algorithmic biases, exacerbated by non-representative datasets, compromise reliability, and “black box” decision-making and LLM inaccuracies erode trust. Furthermore, regulatory gaps in data privacy and liability frameworks complicate adoption. Future integration requires culturally inclusive data curation and hybrid validation frameworks to mitigate biases. Collaboration between clinicians and AI systems must prioritize human oversight, while regulatory harmonization should address data sovereignty and device certification. Incorporating AI literacy into medical curricula and patient education initiatives will foster trust, and industry–institution partnerships can expedite the development of affordable, context-specific tools for global scalability.

The true potential of AI lies not in replacing human expertise, but in augmenting the synergy between artistic intuition and scientific rigor. By prioritizing ethics, equity, and multi-stakeholder collaboration, AI has the potential to democratize access to personalized, safe aesthetic care, transforming cosmetic practice into a discipline that is universally precise and patient-centered.
